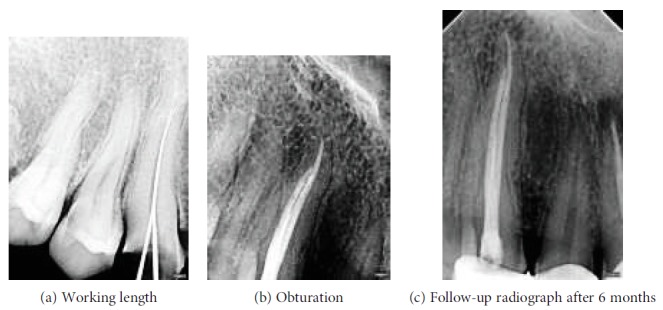# A Case Report on Endodontic Management of a Rare Vertucci Type III Maxillary Canine

**DOI:** 10.1155/2019/4154067

**Published:** 2019-01-29

**Authors:** Hrudi Sundar Sahoo, R. Kurinji Amalavathy, D. Pavani

**Affiliations:** Department of Conservative Dentistry and Endodontics, Sathyabama Dental College, Chennai, Tamil Nadu, India

## Abstract

Success in root canal treatment demands a thorough knowledge of usual root canal anatomy and its variations pertaining to every tooth. Variations in root canal anatomy are often accompanied by complex orientation of pulp tissues making a thorough mechanical and chemical debridement a challenge. Inability to treat such complexities often leads to endodontic failure. Upon a quick review of the literature, it has been noted that very few root canal complexities in maxillary canines have been reported. To be a successful clinician, one must be aware of such rare anatomical instances in maxillary canines. Based on possible branching of the root canal system, root canal configurations of permanent teeth were divided into eight different types by Vertucci. The classification included single to three separate root canals. This case report presents a permanent right maxillary canine which is single rooted having a single canal orifice and a root canal dividing into two canals (buccal and palatal) at the middle third of the root and then joining at the apical third, before exiting with a single apical foramen (Vertucci type III).

## 1. Introduction

Success in root canal treatment demands a thorough knowledge of usual root canal anatomy and its variations pertaining to every tooth. Variations in root anatomy are often accompanied by complex orientation of pulp tissues making a thorough mechanical and chemical debridement a challenge. Inability to treat such complexities often leads to endodontic failure. One of the major reasons for endodontic failure can be missing extra root canals [1]. With advancements in digital imaging, magnification, instrumentation, and disinfection, treating complicated root canal systems has become more predictable.

The root canal systems include an intricate network of pulp tissues that include blood vessels and nerve tissues. Upon review of the literature, a large number of root canal anatomical variations have been reported in human anterior teeth. The possibility of finding two or three root canals in lower anterior teeth can range between 1% and 43%. Among lower anterior teeth, usually the canines are known to have one root and one root canal. Vertucci and Bellizzi and Hartwell had reported that 15% of mandibular canines had two separate root canals with one or two separate exits [2, 3]. Later on, few case reports cited the occurrence of two roots and three root canals, three root canals and two exits, and two separate roots and two root canals [4–6]. On the contrary, the upper canines are usually single canaled and single rooted. A maxillary canine with a single root canal dividing into two separate canals followed by joining of those two canals to the exit at a single foramen is a rare anatomy. Such aberrant root canal anatomy can result from abnormal development during tooth formation.

Based on possible branching of the root canal system, Vertucci classified root canal configurations of permanent teeth into eight different types. The classification included single to three separate root canals [7].

This case report presents a permanent right maxillary canine which is single rooted having a single canal orifice and root canal dividing into two canals (buccal and palatal) at the middle third of the root and then joining at the apical third, before exiting with a single apical foramen (Vertucci type III).

## 2. Case Report

**Figure 1:**
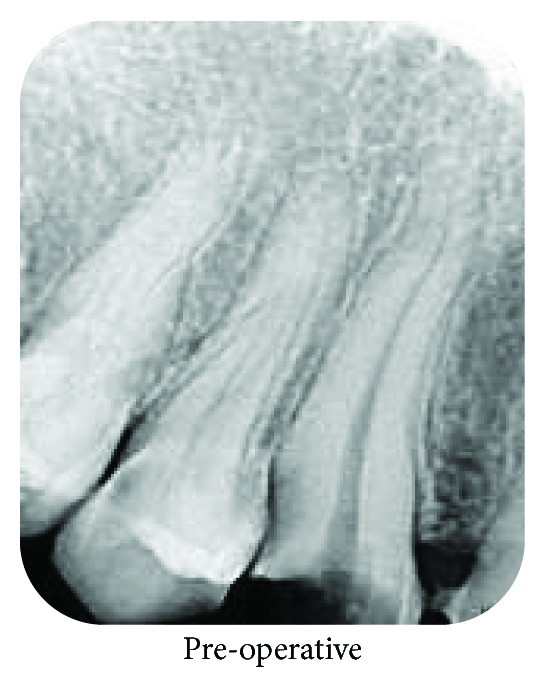


A 33-year-old male patient was referred from a private practitioner to the Department of Conservative Dentistry and Endodontics of Sathyabama Dental College and Hospital, Chennai, with the chief complaint of sensitivity and occasional pain in the left region of upper front teeth. On clinical examination, the patient had a crown-bridge prosthesis spanning from the left upper canine to the right upper canine. Since the crown-bridge prosthesis had a compromised stability, it was removed and an intraoral radiograph in relation to the #12 and #13 region was taken (Figure 1). The radiograph revealed distoproximal dental caries involving enamel, dentin, and pulp of tooth #13. An electric pulp test suggested symptomatic irreversible pulpitis.

In the first visit, under local anesthesia (Lignox 2%; Indoco Remedies Ltd., Mumbai) and rubber dam (Hygienic, Coltene Whaledent) isolation, root canal treatment was initiated in #13. With the help of an endo-access bur (bur type FG-1; Dentsply, USA), an access cavity was made and a single root canal orifice was located. The tentative working length was found to be 26 mm with an apex locator (Root ZX mini; J Morita, Japan). Hand instrumentation (K-files, Mani Inc., Japan) was done till size #50. A copious saline and sodium hypochlorite (3%) irrigation was done during each instrumentation change. Calcium hydroxide (RC-Cal; Prime Dental Ltd., India) was placed as an intracanal medicament. The access cavity was temporized with Cavit (3M ESPE, Germany), and a second visit was scheduled for further management.

**Figure 2:**
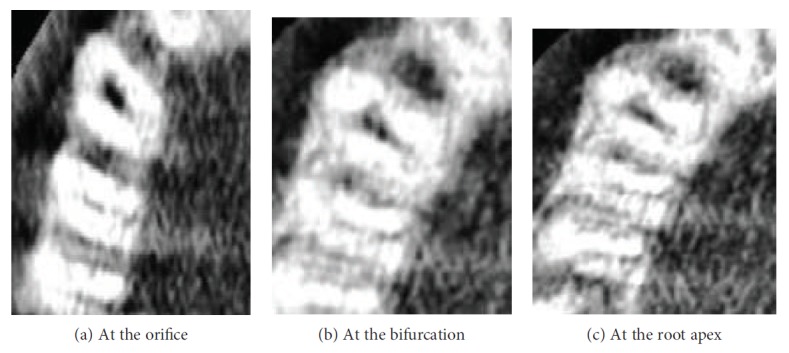


**Figure 3:**
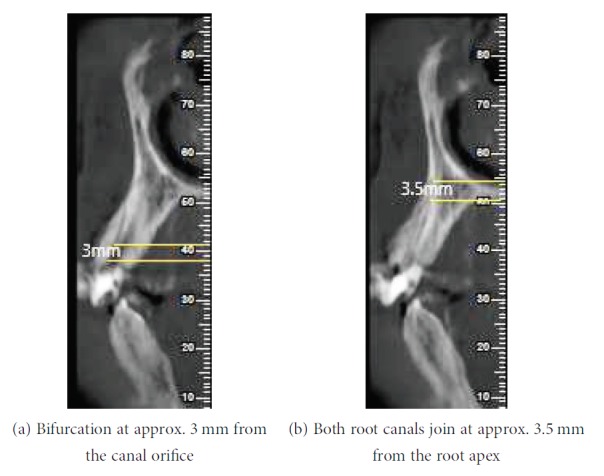


Before the scheduled second visit, the patient reported to the department with severe pain in relation to #13. On reentering into the access cavity, fresh bleeding was noted. Hence, multiple angulated radiographs with two #20-size hand K-files inside the root canal were taken to rule out the presence of any extra root canal. These radiographs were inconclusive of missed canals. According to the AAE and AAOMR Joint Position Statement (2016 update), cone beam computed tomography scanning with a low-field volume, following ALARA principles, was done. On analysing CBCT multiple axial images (Figures 2(a)–2(c)), a second root canal (palatal canal) was seen branching out from the main root canal (buccal canal) at the middle third of the root. The palatal root canal joins the buccal root canal at the apical third, just before the exit suggesting Vertucci type III canal configuration (Figures 3(a) and 3(b)). The palatal canal was negotiated with #10 hand K-files under a dental operating microscope (Seiler Alpha Air 3; St. Louis, USA) at 10x magnification. A working length radiograph was taken to confirm the presence of the palatal canal (Figure 4(a)). After orifice enlargement with a #1 Gates Glidden drill (Mani Inc., Japan), instrumentation was done till #20 hand K-file (Mani Inc., Japan) followed by preparation of the remaining canal using the Self-Adjusting File (SAF; ReDent, Ra'anana, Israel) and the VATEA irrigation pump for chemical debridement with 3.5% sodium hypochlorite during canal preparation.

In the scheduled third visit, the patient was asymptomatic. In view of complicacy of the root canal, the obturation of both root canals (Figure 4(b)) was carried out using thermoplasticized gutta percha (Elements; Sybron Endo, Germany). The follow-up review radiograph (Figure 4(c)) after 6 months revealed no periapical changes, and the patient was found to be asymptomatic.

## 3. Discussion

A few indications of an aberrant root canal anatomy are a modified coronal access, unusual location and size of the canal orifice, and indistinct X-ray images [8]. In this case, multiple angulated digital X-rays failed to provide definitive information of any variation in the root canal system.

Routine radiographs most often fail to indicate additional root canals or any variation in root canal anatomy whereas CBCT has been highly successful in facilitating a better visualization and three-dimensional imaging of such unusual anatomy. Unlike conventional CT scans, CBCT provides higher resolution with reduced radiation dose [9–11]. The principle of “as low as reasonably achievable” (ALARA) was considered, but the necessity and advantage of using CBCT in this case outweighed the risks of additional exposure. Hence, for better understanding of the root canal system, a judicious use of cone beam computed tomography was required in the current case. CBCT of tooth #13 performed in this case confirmed the existence of two canals (one palatal and one buccal) as well as vividly presented the course of these two canals (Vertucci type III). Hence, CBCT definitively helped in the proper diagnosis and improved the treatment success by enabling the description of such a rare anatomic variation precisely.

Çalişkan et al. [12] studied the root canal number, configuration, and ramifications of permanent teeth in a Turkish population. They reported percentage of Vertucci type III [1 -2 -1] as 4.35% whereas Nikhita et al. [13] studied 250 maxillary canines in an Indian population and reported the occurrence of Vertucci type III as 11.6%.

To probe for an additional canal, a tactile examination of all the walls of the major canal was performed with the tip of a precurved scouting hand K-file (size #10). A catch was felt in the palatal surface of the major canal wall. Hence, the presence of a possible canal bifurcation was suspected. Green [14] reported that on deeper penetration into a canal, if an instrument demonstrates eccentric direction, termed directional control, an additional canal should be suspected. With the aid of CBCT axial images, the second root canal orifice was located under a dental operating microscope. The role of a dental microscope in endodontic practice cannot be underestimated as it helped to locate and visualize the second orifice at almost the middle third of the root canal (the point of bifurcation of the main root canal). Such an aberrant anatomy can also be expected to occur in the contralateral maxillary canine. Hence, such findings can also serve as an alert to a clinician while treating the left maxillary canine, if necessary, in the future.

The buccal canal that was in line with the main passage is usually amenable to adequate enlarging and obturation procedures; the preparation and filling of the palatal canal were extremely difficult. Under an endodontic microscope, after scouting and shaping the palatal canal till #20, a self-adjustable file system was used to chemomechanically debride the canal system. The Self-Adjusting File (SAF) (ReDent Nova, Ra'anana, Israel) is a uniquely designed, thin-walled, hollow endodontic file made of a NiTi lattice that is compressible in a canal. The irrigant flow through the hollow file provides chemical debridement with simultaneous enlargement of the canal [15]. Hence, the use of the SAF system in preparing such a complicated root canal system in this case was deemed necessary.

## 4. Conclusion

The root canal system presents a myriad of complexities. While identifying such variations is a challenge itself, cone beam computed tomography was deemed necessary to understand the complexities present in this case. In addition to such an advanced imaging technique, the use of magnification and the advanced canal preparation system like the Self-Adjusting File system helped us deliver a predictable treatment outcome.

## Figures and Tables

**Figure 4:**